# Assessing associated factors for failure of nonoperative management in pediatric blunt liver and spleen injuries: a secondary analysis of the SHIPPs study

**DOI:** 10.1007/s00068-024-02575-y

**Published:** 2024-06-18

**Authors:** Shunichiro Nakao, Morihiro Katsura, Masayuki Yagi, Hiroshi Ogura, Jun Oda

**Affiliations:** 1 Department of Traumatology and Acute Critical Medicine, Osaka University Graduate School of Medicine, 2-15 Yamadaoka, Suita, Osaka 565-0871 Japan; 2 Department of Surgery, Okinawa Chubu Hospital, Uruma, Okinawa Japan; 3 Emergency Medicine and Acute Care Surgery, Matsudo City General Hospital, Matsudo, Chiba Japan

**Keywords:** Nonoperative management, Pediatric blunt liver injury, Pediatric blunt spleen injury

## Abstract

**Purpose:**

The purpose of this study was to describe the characteristics of pediatric patients who underwent nonoperative management (NOM) for blunt splenic and hepatic injuries and to explore factors associated with NOM failure.

**Methods:**

This was a secondary analysis of a multicenter cohort study of pediatric patients with blunt liver and spleen injuries in Japan. Participants included pediatric trauma patients aged 16 years or younger between 2008 and 2019 with NOM, which was defined as no surgery provided within 6 h of hospital arrival. NOM failure, defined as abdominal surgery performed after 6 h of hospital arrival, was the primary outcome. Descriptive statistics were provided and exploratory analysis to assess the associations with outcome using logistic regression.

**Results:**

During the study period, 1339 met our eligibility criteria. The median age was 9 years, with a majority being male. The median Injury Severity Score (ISS) was 10. About 14.0% required transfusion within 24 h, and 22.3% underwent interventional radiology procedures. NOM failure occurred in 1.0% of patients and the in-hospital mortality was 0.7%. Factors associated with NOM failure included age, positive focused assessment with sonography for trauma (FAST), contrast extravasation on computed tomography (CT), severe liver injury, concomitant pancreas injury, concomitant gastrointestinal injury, concomitant mesenteric injury, and ISS.

**Conclusions:**

In our study, NOM failure were rare. Older age, positive FAST, contrast extravasation on CT, severe liver injury, concomitant pancreas injury, concomitant gastrointestinal injury, concomitant mesenteric injury, and higher ISS were suggested as possible risk factors for NOM failure.

**Supplementary Information:**

The online version contains supplementary material available at 10.1007/s00068-024-02575-y.

## Introduction

In pediatric blunt liver and spleen injuries, nonoperative management (NOM) has become the preferred approach in current practice. The guidelines by the German Trauma Society recommended NOM for hemodynamically stable isolated liver and spleen injuries in children, involving close monitoring and preparation for immediate interventional radiology and/or surgical intervention [1]. Guidelines from Level I pediatric trauma centers in the United States suggest that management of pediatric blunt liver and spleen injuries may be based on hemodynamic status rather than injury grade, with unstable patients considered for surgery, urgent embolization, or continued NOM, depending on other injuries and the center's resources [2]. Updated guidelines published in 2023 by the American Pediatric Surgical Association discuss patients exceeding the shock index, pediatric age-adjusted (SIPA) cutoffs, deeming them unstable and more likely to experience NOM failure [3]. However, management of pediatric blunt liver and spleen injuries presents may vary among Europe and the United States in several aspects, as the indication for surgical intervention can differ based on institutional capabilities [4].

Evidence exists regarding associated factors contributing to NOM failure in pediatric blunt liver and spleen injuries. A retrospective study identified NOM failure in pediatric solid organ injury associated with injury severity, presence of multiple organ injuries, and pancreatic injury [5]. Furthermore, a prospective observational study conducted at level I pediatric trauma centers demonstrated associations between NOM failure in pediatric liver and spleen injuries and contrast extravasation on computed tomography (CT), early transfusion, and injury to multiple intra-abdominal organs [6]. A previous systematic review supported that the management of liver and spleen injuries in children should include consideration of the presence of contrast extravasation on CT in addition to the physiologic response [7]. Another analysis in this study group identified that negative focused abdominal sonography for trauma examination was predictive of successful NOM of pediatric blunt liver and spleen injuries [8].

However, due to the infrequent occurrence of NOM failure, there remains insufficient evidence to characterize its determinants in pediatric blunt liver and spleen injuries, underscoring the necessity for individualized risk assessment. The purpose of this study was to describe the characteristics of pediatric patients who underwent NOM for blunt splenic and hepatic injuries and to explore factors associated with NOM failure.

## Methods

### Study design and setting

We performed a secondary analysis of data derived from the previously reported the Splenic and Hepatic Injury in Pediatric Patients (SHIPPs) study, which was a multicenter cohort study in pediatric patients with blunt liver and spleen injury in Japan [9, 10]. The institutional ethics committee of Osaka University Graduate School of Medicine approved this study and waived the requirement for informed consent (approval no. 20129). Given the non-interventional nature of this study, the necessity for individual patient informed consent was waived for this study.

### Participants

We included pediatric blunt trauma patients aged 16 years or younger with liver and splenic injury, who were enrolled in the SHIPPs study from 83 centers between 2008 and 2019 [9, 10]. We excluded patients who had cardiopulmonary arrest on arrival, an abbreviated injury scale (AIS) 6 injury of any body region, a parent or guardian refusal of treatment due to a severe head injury (head AIS 5 +), and a transfer to another hospital within 5 days of admission without required follow-up information. Attempted NOM was defined as no surgery within 6 h after hospital arrival in accordance with a previous study [11]. Therefore, we excluded those with operative management within 6 h.

### Variables

We extracted the following patient data: age, sex, mechanism of injury, vital signs on hospital arrival, results of focused assessment with sonography in trauma (FAST), presence of contrast extravasation on CT, injury site (liver, spleen, or both), injury grade for liver and spleen according to the American Association for the Surgery of Trauma Organ Injury Scale grade (2018 revision), concomitant injury (AIS 3 +) to the head/neck, thorax, or pelvis/lower-extremity, concomitant intra-abdominal injury, injury severity score (ISS), transfusion administration within 24 h, need for interventional radiology, time to surgery from hospital arrival, reason for surgery, and in-hospital mortality. The presence of shock on arrival was defined using SIPA [12]. We classified liver injuries graded IV and V as severe liver injuries, and spleen injuries graded IV and V as severe spleen injuries, based on guidelines from the World Society of Emergency Surgery [13, 14]. The outcome of interest in this study was NOM failure, which was defined as abdominal surgery performed after attempted NOM over 6 h of hospital arrival.

### Statistical methods

Continuous variables are presented as the median and interquartile range (IQR) and categorical variables are presented as the number and percentage. We explored the association between the NOM failure and relevant variables including age, sex, shock on arrival, positive FAST, contrast extravasation on CT, liver injury grade IV or V, spleen injury grade IV or V, concomitant intra-abdominal injuries, and ISS by a univariable logistic regression analysis and calculated odds ratios (ORs) and 95% confidence intervals (CIs). A multivariable logistic regression analysis was planned if the outcome events were observed 20 or more to avoid the risk of overfitting and decreasing the confidence in reported findings [15]. We also described the characteristics in cases with NOM failure and in-hospital mortality. To assess changes in patients’ characteristics and outcomes over time, we divided the research period into early (2008–2013) and late (2014–2019) time periods, then repeated the same analyses. We used Mann–Whitney U test or chi-squared test with Yates' continuity correction to compare these two time periods as needed.

All tests were two-tailed, and P values of < 0.05 were considered to indicate statistical significance. All statistical analyses were performed using R Statistical Software (version 3.6.2; R Foundation for Statistical Computing, Vienna, Austria).

## Results

**Fig. 1:**
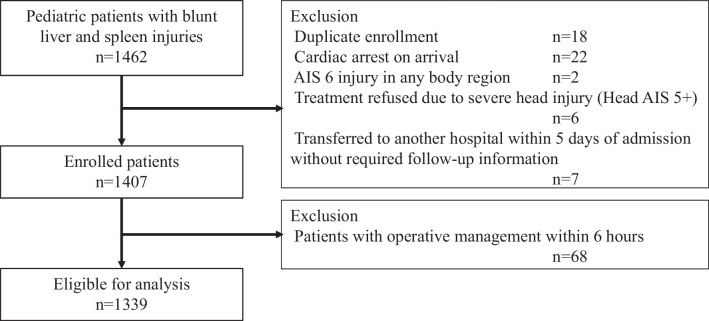
Patient flow

During the study period, the SHIPPs database recorded 1462 pediatric patients with blunt liver and spleen injuries, of which 1339 met our eligibility criteria, as shown in Fig. 1. Characteristics of 83 participating centers were shown in Table S1. In most participating centers, surgeons and interventional radiologists were available whenever needed (89.2% and 80.7%, respectively), while in the remaining centers, their availability was limited. Table 1 summarizes the characteristics of these eligible patients. The median age was 9 years (IQR, 6 to 13) and more than half were male (66.5%). The most common injury mechanisms were pedestrian accidents (24.2%), falls from height or down stairs (22.2%), and bicycle crashes (19.1%). Liver injury was observed in 62.4% of patients, spleen injury in 40.9%, and both liver and spleen injuries in 3.2% of cases. Concomitant kidney injury occurred in 9.5% of patients, pancreas injury in 2.2%, gastrointestinal injury in 0.9%, and mesenteric injury in 0.3%. The median ISS was 10 (IQR, 5 to 18), 14.0% required blood transfusion within 24 h of hospital arrival, and 22.3% underwent interventional radiology procedures. Overall, 1.0% of patients experienced NOM failure, while the in-hospital mortality was 0.7%.

**Table 1:** Patient characteristics in nonoperative management of pediatric blunt liver and spleen injuries

Characteristics	Total (n = 1,339)	
Age, median, Q1-Q3	9	6–13
Male sex, n (%)	891	(66.5)
Mechanism, n (%)		
Car crash	156	(11.7)
Bicycle crash	256	(19.1)
Pedestrian	324	(24.2)
Fall from height/fall down stairs	297	(22.2)
Fall on the ground	104	(7.8)
Sports-related injury	118	(8.8)
Assault/abuse	30	(2.2)
Others	54	(4.0)
Shock on arrival,* n (%)	332	(24.8)
Positive FAST, n (%)	550	(41.1)
Contrast extravasation on CT, n (%)	210	(15.7)
Injury site, n (%)		
Liver injury	835	(62.4)
Spleen injury	547	(40.9)
Both liver and spleen injuries	43	(3.2)
Liver injury grade,** n (%)		
I	171	(12.8)
II	370	(27.6)
III	199	(23.8)
IV	90	(10.8)
V	5	(0.6)
Spleen injury grade,** n (%)		
I	46	(3.4)
II	197	(14.7)
III	180	(32.9)
IV	94	(17.2)
V	30	(5.5)
Concomitant intra-abdominal injury, n (%)	163	(12.2)
Kidney	127	(9.5)
Pancreas	29	(2.2)
Gastrointestinal tract	12	(0.9)
Mesentery	4	(0.3)
Concomitant injury to other body regions, n (%)		
Head/neck	158	(11.8)
Thorax	310	(23.2)
Pelvis/lower-extremity	46	(3.4)
ISS, median, Q1-Q3	10	5–18
Transfusion administration, n (%)	187	(14.0)
Interventional radiology performed, n (%)	299	(22.3)
Nonoperative management failure, n (%)	13	(1.0)
Length of hospital stay in days, median, Q1-Q3	12	7–19
In-hospital mortality, n (%)	10	(0.7)

FAST, Focused Assessment with Sonography for Trauma; CT, Computed Tomography; ISS, Injury Severity Score

^*^Shock was defined based on shock index, pediatric age-adjusted (SIPA) above cutoff

^**^The American Association for the Surgery of Trauma Organ Injury Scale grade (2018 revision) was used

Table 2 presents the results of univariate logistic regression analyses, indicating the odds ratios of each variable for NOM failure. Factors associated with NOM failure included age, positive FAST, contrast extravasation on CT, liver injury grade IV or V, concomitant pancreas injury, concomitant gastrointestinal injury, concomitant mesenteric injury, and ISS. Due to the limited number of outcomes, multivariable analysis was not conducted according to the initial plan.

**Table 2:** Odds ratios of each variable for NOM failure through univariate logistic regression analyses

	NOM failure			
	%	n/N	OR (95% CI)	*P* value
Age	-	-	1.16 (1.01 to 1.35)	0.043
Sex				
Male	1.1	(10/891)	1.68 (0.51 to 7.54)	0.430
Female	0.7	(3/448)	Reference	-
Shock on arrival*				
(+)	1.2	(4/332)	1.31 (0.35 to 4.06)	0.652
(-)	0.9	(9/978)	Reference	-
Positive FAST				
(+)	1.8	(10/550)	4.85 (1.48 to 21.71)	0.017
(-)	0.4	(3/789)	Reference	
Contrast extravasation on CT				
(+)	2.9	(6/210)	5.60 (1.67 to 19.60)	0.005
(-)	0.5	(5/957)	Reference	
Severe liver injury**				
(+)	4.2	(4/95)	6.03 (1.61 to 18.91)	0.003
(-)	0.7	(9/1244)	Reference	-
Severe spleen injury**				
(+)	1.6	(2/124)	1.79 (0.28 to 6.78)	0.450
(-)	0.9	(11/1215)	Reference	-
Concomitant kidney injury				
(+)	0.8	(1/127)	0.79 (0.04 to 4.08))	0.825
(-)	1.0	(12/1212)	Reference	-
Concomitant pancreas injury				
(+)	10.3	(3/29)	15.00 (3.23 to 52.49)	< 0.001
(-)	0.8	(10/1310)	Reference	-
Concomitant gastrointestinal tract injury				
(+)	8.3	(1/12)	9.96 (1.19 to 83.40)	0.034
(-)	0.9	(12/1327)	Reference	-
Concomitant mesenteric injury				
(+)	25.0	(1/4)	36.75 (1.75 to 311.64)	0.002
(-)	0.9	(12/1335)	Reference	-
ISS	-	-	1.07 (1.03 to 1.11)	< 0.001

OR, odds ratio; CI, confidence interval; FAST, Focused Assessment with Sonography for Trauma; CT, Computed Tomography; ISS, Injury Severity Score

^*^Shock was defined based on shock index, pediatric age-adjusted (SIPA) above cutoff

^**^The American Association for the Surgery of Trauma Organ Injury Scale grade (2018 revision) IV and V were considered as severe injuries

Table 3 outlines the characteristics of the 13 patients who experienced NOM failure. Among them, 30.8% presented with shock upon hospital arrival, while 46.2% exhibited contrast extravasation on CT scans. Out of 13 patients with NOM failure, 10 patients (76.9%) had liver injury, 4 (30.8%) had spleen injury and 1 (7.7%) had both. Interventional radiology was required for 76.9% of these cases. The median time from hospital arrival to surgery was 20 h (IQR, 10 to 123.5), with active bleeding being the most common reason for surgical intervention (53.8%). Of these 7 cases of NOM failure due to active bleeding, 4 underwent interventional radiology as the initial intervention. In-hospital mortality among NOM failure cases stood at 15.4%, with 2 out of the 13 cases of NOM failure. The causes of death in both cases were hemorrhagic shock due to liver/spleen injury, with one case also indicating traumatic brain injury as an additional cause of death. Table 4 summarizes patient characteristics in the 10 cases resulting in in-hospital mortality, with traumatic brain injury identified as the primary cause of death in 70.0% of cases. We did not see significant changes in patients’ characteristics between the early (2008–2013) and late (2014–2019) time periods (Table S2). Proportions of interventional radiology and NOM failure did not significantly change over time in our study.

**Table 3:** Patient characteristics in NOM failure

Characteristics	NOM failure (n=13)	
Age, median, Q1-Q3	15	7-16
Male sex, n (%)	10	(76.9)
Shock on arrival,* n (%)	4	(30.8)
Positive FAST, n (%)	10	(76.9)
Contrast extravasation on CT, n (%)	6	(46.2)
Injury site, n (%)		
Liver injury	10	(76.9)
Spleen injury	4	(30.8)
Both liver and spleen injuries	1	(7.7)
Liver injury grade,** n (%)		
I	0	(0.0)
II	1	(7.7)
III	5	(38.5)
IV	3	(23.1)
V	1	(7.7)
Spleen injury grade,** n (%)		
I	0	(0.0)
II	2	(15.4)
III	0	(0.0)
IV	1	(7.7)
V	1	(7.7)
Concomitant intra-abdominal injury, n (%)	4	(30.8)
Kidney	1	(7.7)
Pancreas	3	(23.1)
Gastrointestinal tract	1	(7.7)
Mesentery	1	(7.7)
ISS, median, Q1-Q3	20	16-41
Transfusion administration, n (%)	10	(76.9)
Interventional radiology performed, n (%)	6	(46.2)
Time to surgery from hospital arrival, hours, median, IQR	20	10-123.5
Reason for surgery, n (%)		
Active hemorrhage	7	(53.8)
Peritonitis/infection	2	(15.4)
Bile leak/biloma	1	(7.7)
Duodeno-pancreatic injury	1	(7.7)
In-hospital mortality, n (%)	2	(15.4)

FAST, Focused Assessment with Sonography for Trauma; CT, Computed Tomography; ISS, Injury Severity Score

*Shock was defined based on shock index, pediatric age-adjusted (SIPA) above cutoff.

**The American Association for the Surgery of Trauma Organ Injury Scale grade (2018 revision) was used.

**Table 4:** Patient characteristics in cases of mortality

Characteristics	Mortality (n=10)	
Age, median, Q1-Q3	9.5	7-14.75
Male sex, n (%)	7	(70.0)
Shock on arrival,* n (%)	5	(50.0)
Positive FAST, n (%)	4	(40.0)
Contrast extravasation on CT, n (%)	3	(30.0)
Injury site, n (%)		
Liver injury	4	(40.0)
Spleen injury	7	(70.0)
Both liver and spleen injuries	1	(10.0)
Liver injury grade,** n (%)		
I	0	(0.0)
II	4	(40.0)
III	1	(10.0)
IV	2	(20.0)
V	0	(0.0)
Spleen injury grade,** n (%)		
I	0	(0.0)
II	2	(20.0)
III	1	(10.0)
IV	0	(0.0)
V	1	(10.0)
Concomitant intra-abdominal injury, n (%)	3	(30.0)
Kidney	2	(20.0)
Pancreas	1	(10.0)
Gastrointestinal tract	0	(0.0)
Mesentery	0	(0.0)
ISS, median, Q1-Q3	43	38.75-50
Transfusion administration, n (%)	10	(100.0)
Interventional radiology performed, n (%)	3	(30)
Nonoperative management failure, n (%)	2	(20.0)
Cause of death,*** n (%)		
Traumatic brain injury	7	(70.0)
Hemorrhage shock due to liver/spleen injury	2	(20.0)
Hemorrhage shock due to other injury	1	(10.0)
Respiratory failure	1	(10.0)

FAST, Focused Assessment with Sonography for Trauma; CT, Computed Tomography; ISS, Injury Severity Score

*Shock was defined based on shock index, pediatric age-adjusted (SIPA) above cutoff.

**The American Association for the Surgery of Trauma Organ Injury Scale grade (2018 revision) was used.

***In one case, both traumatic brain injury and hemorrhagic shock due to liver/spleen injury were recorded as causes of death.

## Discussion

In this study, we investigated the characteristics of pediatric patients who underwent NOM for blunt liver and spleen injuries, as well as explored factors associated with NOM failure. We observed a low incidence of NOM failure among pediatric patients with blunt liver and spleen injuries with only 1.0% of patients experiencing NOM failure, suggesting that NOM is a safe and effective approach for the majority of pediatric patients with these injuries, as demonstrated in previous literature [5, 6, 16]. In our study, we identified several potential risk factors associated with NOM failure in pediatric blunt liver and spleen injuries, including older age, positive FAST, contrast extravasation on CT, severe liver injury, concomitant pancreas injury, concomitant gastrointestinal injury, concomitant mesenteric injury, and higher ISS. Although our findings necessitate further investigation, they could help identifying patients at risk of NOM failure and determining the optimal timing for surgical intervention.

While some of these factors were consistent with previous literature, others showed discrepancies. While previous study on factors associated with NOM failure in pediatric blunt abdominal injury suggested NOM failure was not associated with age but was associated with pancreatic injury, both older age and concomitant pancreas injury were associated with higher incidence of NOM failure in our study [17]. Another cohort study showed that negative FAST and negative SIPA in the emergency department could be predictive of successful NOM of pediatric blunt liver and spleen injuries [8]. In our study, a positive FAST was associated with NOM failure, consistent with previous findings, while shock on arrival using SIPA was not associated with NOM failure. This discrepancy may arise from successful resuscitation in some cases upon arrival, with most NOM failure cases not presenting with shock initially but developing it later. Alternatively, reasons for surgery in NOM failure cases may not necessarily be due to active bleeding. Contrast extravasation on CT was also associated with NOM failure, as suggested in a previous systematic review [7]. A previous study found minimal transition to surgery or interventional radiology when contrast extravasation is detected, with nonoperative treatment success prevailing [18]. However, the authors of this study cautioned that overall clinical assessment remains crucial in determining the need for intervention.

Severe liver injury was associated with NOM failure, while severe spleen injury was not associated in our study. This could be attributed to the recommendation of angioembolization as an alternative to splenectomy in pediatric spleen injury, as outlined in guidelines [1, 2, 19]. Most patients with NOM failure had liver injury in our study, including one patient with bile leak/biloma as a reason for surgery. Prior single-center retrospective observational studies have indicated high success rates of NOM in pediatric blunt liver injuries, regardless of the grade [20, 21]. However, our study's findings suggest the need for caution when considering NOM for high-grade liver injuries. Previous literature indicated that factors such as ISS, multiplicity of injured organs, and pancreatic injuries were associated with NOM failure in pediatric solid organ injury [5]. This literature also highlighted common reasons for NOM failure, including peritonitis and hollow organ injury, as well as shock and persistent hemorrhage. Our study yielded similar results, although concomitant kidney injury was not associated with NOM failure. The distribution of reasons for surgery or NOM failure was similar as well. Based on the proportions of transfusion administration within 24 h and interventional radiology performed in our study, these procedures were successfully provided to hemodynamically stabilize as described in guidelines [1–3, 6].

### Limitations

There are several limitations in this study. First, the SHIPPs study was a multicenter retrospective cohort study that did not use a specific protocol for treatment strategies for pediatric trauma. As reported in previous studies, there may be variation in treatment strategies from facility to facility and even within facilities, which may have affected the results [22, 23]. Second, NOM failure was rare, and although this was a multicenter study, NOM failure was only 0.8%, so multivariate analysis was not possible. In addition, the results may not be generalizable due to differences in emergency systems, such as accessibility to CT scans and angiography in trauma care. Our findings may reflect these unique Japanese practice patterns diverging from the European and American standards, emphasizing the need to consider these differences when interpreting the results. Although surgeons and interventional radiologists were available anytime needed in most participating centers, centers with limited access to interventional radiology may be more inclined towards operative management, potentially impacting the outcomes of NOM. While we were unable to conduct multivariable analysis to adjust for confounding, directing attention towards these potential associated factors, alongside close hemodynamic monitoring, may be valuable during NOM in pediatric blunt liver and spleen injuries.

## Conclusions

Among pediatric blunt liver and spleen injuries who underwent NOM, cases of NOM failure were rare. In our study, older age, positive FAST, contrast extravasation on CT, severe liver injury, concomitant pancreas injury, concomitant gastrointestinal injury, concomitant mesenteric injury, and higher ISS were suggested as possible risk factors for NOM failure. In addition to close hemodynamic monitoring during NOM in pediatric blunt liver and spleen injuries, focusing on these factors may be valuable to improve pediatric trauma care.

## Supplementary Information

Below is the link to the electronic supplementary material.

Supplementary file1 (DOCX 42 KB)

## Data Availability

The data that support the findings of this study are available from the SHIPPs study group, but the availability of these data is restricted.
