# Supplementary material for: Assessing associated factors for failure of nonoperative management in pediatric blunt liver and spleen injuries: a secondary analysis of the SHIPPs study
Source: Eur J Trauma Emerg Surg. 2024 Jun 18;50(5):2249–57. doi: 10.1007/s00068-024-02575-y (PMC11599619; doi:10.1007/s00068-024-02575-y)
Supplement: Supplementary file 1 — Supplementary file1 (DOCX 42 KB) [file 68_2024_2575_MOESM1_ESM.docx]

**Tables**

**Table S1. Characteristics of participating institutions**

| **Characteristics** | **n=83** | |
| --- | --- | --- |
| Type of institution, n (%) |  |  |
| University hospital | 26 | (31.3) |
| Community hospital (adult center) | 45 | (54.2) |
| Children's hospital | 8 | (9.6) |
| Mixed adult/pediatric center | 4 | (4.8) |
| Surgeons available anytime needed | 74 | (89.2) |
| Interventional radiologists available anytime needed | 67 | (80.7) |

**Table S2. Patient characteristics in nonoperative management of pediatric blunt liver and spleen injuries in early and late phases**

| **Characteristics** | **2008 – 2013**  **n=626** | | **2014 – 2019**  **n=713** | | **P value** |
| --- | --- | --- | --- | --- | --- |
| Age, median, Q1-Q3 | 9 | 6-13 | 9 | 6-13 | 0.861 |
| Male sex, n (%) | 410 | (65.5) | 481 | (67.5) | 0.482 |
| Mechanism, n (%) |  |  |  |  | 0.052 |
| Car crash | 66 | (10.5) | 90 | (12.6) |  |
| Bicycle crash | 125 | (20.0) | 131 | (18.4) |  |
| Pedestrian | 159 | (25.4) | 165 | (23.1) |  |
| Fall from height/fall down stairs | 137 | (21.9) | 160 | (22.4) |  |
| Fall on the ground | 46 | (7.3) | 58 | (8.1) |  |
| Sports-related injury | 43 | (6.9) | 75 | (10.5) |  |
| Assault/abuse | 16 | (2.6) | 14 | (2.0) |  |
| Others | 34 | (5.4) | 20 | (2.8) |  |
| Shock on arrival,* n (%) | 155 | (25.7) | 177 | (25.1) | 0.856 |
| Positive FAST, n (%) | 266 | (42.5) | 284 | (39.8) | 0.352 |
| Contrast extravasation on CT, n (%) | 99 | (18.0) | 111 | (18.0) | 0.999 |
| Injury site, n (%) |  |  |  |  | 0.463 |
| Liver injury | 396 | (63.3) | 439 | (61.6) |  |
| Spleen injury | 247 | (39.5) | 300 | (42.1) |  |
| Both liver and spleen injuries | 17 | (2.7) | 26 | (3.6) |  |
| Liver injury grade,** n (%) |  |  |  |  | 0.257 |
| I | 75 | (18.9) | 96 | (21.9) |  |
| II | 168 | (42.4) | 202 | (46.0) |  |
| III | 100 | (25.3) | 99 | (22.6) |  |
| IV | 51 | (12.9) | 39 | (8.9) |  |
| V | 2 | (0.5) | 3 | (0.6) |  |
| Spleen injury grade,** n (%) |  |  |  |  | 0.780 |
| I | 19 | (7.7) | 27 | (9.0) |  |
| II | 89 | (36.0) | 108 | (36.0) |  |
| III | 84 | (34.0) | 96 | (32.0) |  |
| IV | 39 | (15.8) | 55 | (18.3) |  |
| V | 16 | (6.5) | 14 | (4.7) |  |
| Concomitant intra-abdominal injury, n (%) | 82 | (13.1) | 81 | (11.4) | 0.375 |
| Kidney | 66 | (10.5) | 61 | (8.6) | 0.252 |
| Pancreas | 16 | (2.6) | 13 | (1.8) | 0.465 |
| Gastrointestinal tract | 4 | (0.6) | 8 | (1.1) | 0.519 |
| Mesentery | 0 | (0.0) | 4 | (0.6) | 0.169 |
| Concomitant injury to other body regions, n (%) |  |  |  |  |  |
| Head/neck | 78 | (12.5) | 80 | (11.2) | 0.537 |
| Thorax | 150 | (24.0) | 160 | (22.4) | 0.553 |
| Pelvis/lower-extremity | 19 | (3.0) | 27 | (3.8) | 0.546 |
| ISS, median, Q1-Q3 | 10 | 6-19 | 9 | 5-17 | 0.266 |
| Transfusion administration, n (%) | 88 | (14.1) | 99 | (13.9) | 0.991 |
| Interventional radiology performed, n (%) | 141 | (22.5) | 158 | (22.2) | 0.925 |
| Nonoperative management failure, n (%) | 5 | (0.8) | 8 | (1.1) | 0.747 |
| Length of hospital stay in days, median, Q1-Q3 | 13 | 8-20- | 11 | 7-18- | 0.001 |
| In-hospital mortality, n (%) | 8 | (1.3) | 2 | (0.3) | 0.564 |

FAST, Focused Assessment with Sonography for Trauma; CT, Computed Tomography; ISS, Injury Severity Score

*Shock was defined based on shock index, pediatric age-adjusted (SIPA) above cutoff.

**The American Association for the Surgery of Trauma Organ Injury Scale grade (2018 revision) was used.

P values were calculated with Mann-Whitney U test or chi-squared test with Yates' continuity correction as needed.

**Table S3. Odds ratios of each variable for NOM failure through univariate logistic regression analyses in early and late phases**

|  | **2008 – 2013 (n=626)** | | | **2014 – 2019 (n=713)** | | |
| --- | --- | --- | --- | --- | --- | --- |
|  | **n/N** | **OR (95% CI)** | **P value** | **n/N** | **OR (95% CI)** | **P value** |
| Age | - | 0.98 (0.79-1.20) | 0.826 | - | 1.38 (1.11-1.85) | 0.012 |
| Sex  Male  Female | 4/410  1/216 | 2.12 (0.31-41.58)  Reference | 0.503 | 6/481  2/232 | 1.45 (0.33-9.97)  Reference | 0.649 |
| Shock on arrival*  (+)  (-) | 2/155  3/449 | 1.94 (0.25-11.83)  Reference | 0.469 | 2/177  6/529 | 1.00 (0.15-4.37)  Reference | 0.996 |
| Positive FAST  (+)  (-) | 2/266  3/360 | 0.90 (0.12-5.48)  Reference | 0.910 | 8/284  0/429 | NA  Reference | NA |
| Contrast extravasation on CT  (+)  (-) | 2/99  1/451 | 9.28 (0.88-200.84)  Reference | 0.070 | 4/111  4/506 | 4.69 (1.09 -20.12)  Reference | 0.031 |
| Severe liver injury**  (+)  (-) | 2/53  3/573 | 7.45 (1.21-45.62)  Reference | 0.030 | 2/42  6/671 | 5.54 (1.08-28.34)  Reference | 0.040 |
| Severe spleen injury**  (+)  (-) | 0/55  5/571 | NA  Reference | NA | 2/69  6/644 | 3.17 (0.46-14.09)  Reference | 0.162 |
| Concomitant kidney injury  (+)  (-) | 0/66  5/560 | NA  Reference | NA | 1/61  7/652 | 1.54 (0.08-8.83))  Reference | 0.691 |
| Concomitant pancreas injury  (+)  (-) | 1/16  4/610 | 10.10 (1.06-95.85)  Reference | 0.044 | 2/13  6/700 | 21.03 (3.81-116.00)  Reference | <0.001 |
| Concomitant gastrointestinal tract injury  (+)  (-) | 1/4  4/622 | 51.50 (2.28-524.00)  Reference | 0.002 | 0/8  8/705 | NA  Reference | NA |
| Concomitant mesenteric injury  (+)  (-) | 0/0  5/626 | NA  Reference | NA | 1/4  7/709 | 33.43 (1.55-301.76)  Reference | 0.004 |
| ISS | - | 1.05 (0.98-1.11) | 0.099 | - | 1.08 (1.03-1.13) | 0.001 |

OR, odds ratio; CI, confidence interval; FAST, Focused Assessment with Sonography for Trauma; CT, Computed Tomography; ISS, Injury Severity Score, NA, not applicable

*Shock was defined based on shock index, pediatric age-adjusted (SIPA) above cutoff.

**The American Association for the Surgery of Trauma Organ Injury Scale grade (2018 revision) IV and V were considered as severe injuries.
